# Molecular evolution of type VI intermediate filament proteins

**DOI:** 10.1186/1471-2148-7-164

**Published:** 2007-09-13

**Authors:** Dominique Guérette, Paul A Khan, Pierre E Savard, Michel Vincent

**Affiliations:** 1 CREFSIP and Département de médecine, Pavillon Charles-Eugène-Marchand, Université Laval, Québec, G1K 7P4, Canada; 2 Unité de recherche en pédiatrie, Centre de recherche du CHUL, Université Laval, Québec, G1V 4G2, Canada; 3 Unité de recherche en Neurosciences, Centre de recherche du CHUL, Université Laval, Québec, G1V 4G2, Canada

## Abstract

**Background:**

Tanabin, transitin and nestin are type VI intermediate filament (IF) proteins that are developmentally regulated in frogs, birds and mammals, respectively. Tanabin is expressed in the growth cones of embryonic vertebrate neurons, whereas transitin and nestin are found in myogenic and neurogenic cells. Another type VI IF protein, synemin, is expressed in undifferentiated and mature muscle cells of birds and mammals. In addition to an IF-typical α-helical core domain, type VI IF proteins are characterized by a long C-terminal tail often containing distinct repeated motifs. The molecular evolution of type VI IF proteins remains poorly studied.

**Results:**

To examine the evolutionary history of type VI IF proteins, sequence comparisons, BLAST searches, synteny studies and phylogenic analyses were performed. This study provides new evidence that tanabin, transitin and nestin are indeed orthologous type VI IF proteins. It demonstrates that tanabin, transitin and nestin genes share intron positions and sequence identities, have a similar chromosomal context and display closely related positions in phylogenic analyses. Despite this homology, fast evolution rates of their C-terminal extremity have caused the appearance of repeated motifs with distinct biological activities. In particular, our *in silico *and *in vitro *analyses of their tail domain have shown that (avian) transitin, but not (mammalian) nestin, contains a repeat domain displaying nucleotide hydrolysis activity.

**Conclusion:**

These analyses of the evolutionary history of the IF proteins fit with a model in which type VI IFs form a branch distinct from NF proteins and are composed of two major proteins: synemin and nestin orthologs. Rapid evolution of the C-terminal extremity of nestin orthologs could be responsible for their divergent functions.

## Background

The intermediate filament (IF) family is composed of more than 70 genes that are expressed in a tissue- and developmental stage- specific manner in metazoan cells [1-3]. All IF proteins exhibit a tripartite structure comprising a central α-helical core domain flanked by globular head and tail regions [4]. Members of the IF family are grouped together in a class of nuclear proteins (lamins: type V) along with four or five classes of cytoplasmic proteins (types I-IV, VI) depending on the criteria used for their classification [3-6]. Keratins represent the first two classes (types I and II) of IF proteins and they are obligatory heteropolymers. Keratin genes are the most abundant IF family members. In humans, they are clustered on chromosomes 17q21 (type I) and 12q13 (type II) [7]. Vimentin, desmin, peripherin and GFAP form type III IF proteins that can assemble in filaments on their own, or in combination with type IV and type VI IF proteins. Neuronal IF proteins comprise NF-L (light), NF-M (medium), NF-H (heavy) neurofilament protein subunits that along with α-internexin are classified as type IV IF proteins.

Upon its identification in 1990, nestin was designated as the prototype of a new IF protein group (type VI) because it did not fall clearly into any of the previously described types [6]. Some debate arose on this classification since nestin gene structure is closely related to the neurofilament branch in having two of its three intron positions in common with NF genes [8]. Accordingly, it had been proposed to re-classify nestin as a type IV IF protein [9]. However, the low level of sequence similarity of the α-helical region of nestin and NF proteins as well as the presence of a third intron in the nestin gene constitute strong arguments in favor of its classification as a distinct type [6,8,10]. Furthermore, the discovery of tanabin in *Xenopus laevis *a few years later led to the proposal that this tanabin protein could be the prototype of a different IF type (type VII) because of the lack of significant sequence similarities with other IF proteins [11]. Shortly after nestin and tanabin were sequenced, the gene structures of synemin [12,13] and transitin [14] were also described. According to their sequence similarities, tanabin was then grouped with transitin, paranemin (a splice variant of transitin), synemin and nestin as type VI IF proteins. All these proteins are distinguished by a long C-terminal extremity and by the fact that they cannot self-form into filaments. Rather, they need other IF proteins to build filamentous structures [10,15].

Tanabin is specific to amphibians, transitin to birds and nestin to mammals. Tanabin is expressed during neurulation of *X. laevis *and its function is not well understood [11]. Transitin and nestin are transiently expressed in myogenic and neurogenic cells of birds and mammals, respectively [6,16-20]. Chicken transitin is co-expressed with vimentin in proliferating myoblasts and is associated for a short period of time with desmin at the Z line during muscle differentiation [17]. Transitin expression persists in the smooth muscle cells of elastic arteries and in Purkinje fibers where it is expressed in association with vimentin [21]. Its expression is also induced in activated Müller glial cells of chicken retinas following acute retinal damage [22]. Paranemin, a splice variant of transitin [14], is important for the formation of an extended IF network when co-transfected with desmin in SW13 cells [23]. Recently, transitin has been shown to play an important role in determining the intracellular localization of Numb in mitotic neuroepithelial cells [24]. In mammals, nestin expression is induced in certain tumors as well as in regenerating skeletal muscles [25,26]. In addition, nestin is implicated in vimentin intermediate filament disassembly during mitosis [27] and is a survival determinant through cdk5 regulation in oxidant-induced cell death [28]. All these observations suggest that both transitin and nestin have important and distinct functions in various cell types during embryonic development and in tissue regeneration in adults.

Despite a low level of sequence identity, the large tail domains of nestin and transitin display some similarities including the presence of highly charged glutamate-rich stretches and of repeated motifs prone to α-helicity. The tail domain of transitin contains a motif comprised of more than 50 leucine zipper-like heptad repeats (HR domain) of the consensus sequence LQVEHGD [29] whereas that of nestin features an 11-amino acid repeat motif whose number of repetitions varies according to the species [6]. Synemin is another type VI IF protein expressed in developing and adult skeletal muscles of both birds and mammals [30-32]. Different studies report that interaction of the long C-terminal tail of synemin with other cytoskeletal components may be a key component linking myofibrillar Z lines to costameres in skeletal muscle cells [33,34].

The molecular evolution of type VI IF proteins remains poorly studied. As already mentioned, the common denominator shared by all IF proteins is the presence of an α-helical region involved in filament assembly. Two prototypes of cytoplasmic IF proteins, defined by the presence or absence of a long lamin-like coil 1b within the α-helical domain, seem to parallel metazoan phylogeny. The first prototype has the long coil 1b subdomain and often a lamin homology segment in its tail domain. It has been documented for 12 protostomic phyla [35,36] and an hemichordate, although the "long" coil 1b is shortened by 11 residues in the latter [36]. The second prototype, restricted to the chordates, contains a coil 1b shortened by 42 residues and lack a lamin homology segment. Following a 42 residue deletion that occured at the origin of the chordate branch, type I-III- IF proteins were established by duplication events and sequence drift. The genes encoding type IV NF proteins have different intron positions than do type I-III genes. They were proposed to be derived from retrotransposition of an intron-less intermediate followed by the acquisition of new introns [37] but this hypothesis has been recently challenged by the documentation of a fish gene combining type I-III intron positions with type IV intron positions [38].

To analyze the evolution of genes encoding type VI IF proteins, sequence comparison, BLAST searching, synteny studies and phylogenic analysis were performed with members of this group. This study provides new evidence that tanabin, transitin and nestin are indeed orthologous type VI IF proteins. These proteins possess significant diversity in composition of their long C-terminal tails that likely provides them with different, but specific functions in myogenic and neurogenic cells of developing vertebrate systems. In particular, *in silico *and *in vitro *analyses provide evidence that the C-terminal extremity of (avian) transitin, but not that of (mammalian) nestin, contains a repeat domain displaying nucleotide hydrolysis activity.

## Results and discussion

### 1-Overview of type VI IF proteins

The cDNA sequence of tanabin from *X. laevis *has been published [11], but the organization of its gene structure is not known. In order to analyze the evolution of type VI IFs, we first determined the exon/intron structure of the tanabin gene of *X. tropicalis *using JGI portal v.4.1. As tanabin was first described in *X. laevis *[11], a BLASTp search was made to determine the putative ortholog of tanabin in *X. tropicalis*. The protein sequence of tanabin from *X. laevis *(tanabin-xl) was used as the query sequence in BLASTp searching carried out at the JGI portal against the *X. tropicalis *genome assembly v4.1. A sequence named fgenesh1_pg.C of 1868 bp was found to be 67% identical to tanabin-xl. Tanabin from *X. tropicalis *(tanabin-xt) is 1970 amino acids (aa) long and possesses the typical α-helical rod domain of IF proteins and a long C-terminal tail of more than 1400 aa. Tanabin-xl and tanabin-xt share more than 90% sequence similarity in their IF rod domain (data not shown).

**Figure 1:**
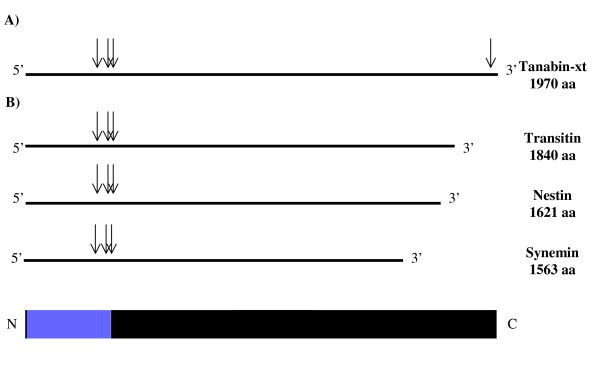
**Schematic representation of the position of introns in type VI intermediate filament genes**. **A) **The arrows along the tanabin cDNA diagram indicate the predicted positions of introns in the tanabin gene deduced from the genomic sequence of *X. tropicalis *(v4.1). **B) **The arrows indicate the location of introns in nestin [6], transitin [14] and synemin [13]. An IF protein diagram is illustrated at the bottom of the figure for orientation purposes. The blue box represents the α-helical rod domain.

The mRNA sequence encoding for tanabin-xt was then compared with the genomic sequence of *X. tropicalis *(assembly v4.1.) to determine intron boundaries (Fig. 1a). The genomic sequence of tanabin-xt revealed the presence of 5 exons of 804 bp, 125 bp, 65 bp, 4848 bp and 71 bp as well as 4 introns of 4610 bp, 3715 bp, 499 bp and 6593 bp, respectively. The first three exons encode for the IF core domain and the last two for the C-terminal domain. Comparison of the gene structure of tanabin with that of the other type VI family members (nestin, transitin, synemin) is illustrated in Figure 1b. All genes share 3 identical intron positions within the 5' portion of the gene and, as already observed [8,39], the positions of the two most upstream introns are also shared by the NF genes. The IF rod domain of type VI IF proteins is thus encoded by three exons. Our analysis also shows that tanabin has a supplementary intron located at the 3'end of the gene (Fig. 1A).

The protein sequences of type VI IF proteins were compared using BL2SEQ on the NCBI web site to verify the level of similarity in their IF rod domain and their C-terminal tail (Table 1). Tanabin-xt and transitin share 51% sequence identity in their rod domain, which is the most important level found among type VI IF proteins. Transitin exhibits 44% identity with human nestin in this domain, a little lower than the usual 50% observed in IF proteins of the same group. The overall level of sequence identity in the carboxy-terminal domain of the proteins is lower at around 20%.

**Table 1:** Protein sequence identity of the alpha helical rod domain and the C-terminal tail of type VI Ifs. Protein sequences deduced from available sequenced genomes present in NCBI and JGI genome assembly v4.1. were analyzed using BL2SEQ searching on the NCBI server. Sequence identities are presented on the table and --- means that no sequence identity was found in these regions of the proteins. Chicken transitin: X80877, human nestin: NM_006617, tanabin-xt: 186291 and human synemin: CAC83859

	**Sequence identity(%) Rod domain**	**Sequence identity(%)C-terminal**
**Transitin vs Tanabin-xt**	51	19
**Transitin vs Nestin**	44	24
**Tanabin-xt vs Nestin**	26	19
**Transitin vs Synemin**	31	---
**Tanabin-xt vs Synemin**	24	---
**Nestin vs Synemin**	30	---

**Figure 2:**
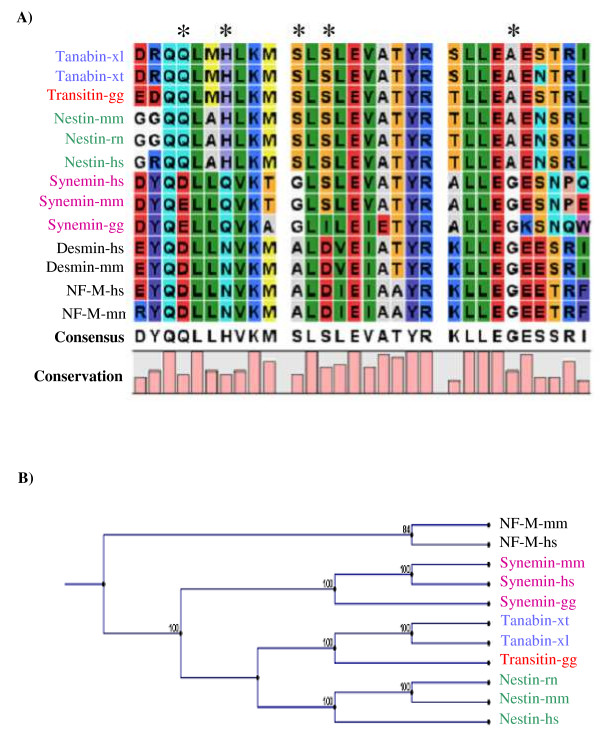
**Evolution of type VI IF proteins**. **A) **Amino acid sequence alignment of the helix termination motif at the end of segment 2B of the α-helical rod domain of type VI IF proteins, desmin and NF-M from different vertebrate species. Asterisks represent amino acid residues specifically conserved in tanabin, transitin and nestin. The consensus sequence deduced from this multiple alignment is indicated at the bottom of the figure. The histogram indicates the level of residue conservation (from 0 to 100%) at each position. **B) **A phylogenetic tree was constructed based on the protein sequences of type IV NF-M and of type VI tanabin, transitin, nestin and synemin from different species using neighbor-joining methods (CLCbio software; 100 bootstraps). A closer evolutionary relationship is noticed between tanabin, transitin and nestin. The bootstrap values are shown at the nodes. xl, *Xenopus laevis*; xt, *Xenopus tropicalis*; gg, *Gallus gallus*; rn, *Rattus norvegicus*; mm, *Mus musculus*; hs, *Homo sapiens*.

A well conserved sequence among IF chains is the helix termination motif at the end of segment 2B of the α-helical rod domain. The consensus sequence for this motif is: E-Y-Q-X-L-L-D/N-V-K-X-R/A-L-D/E-X-E-I-A-T-Y-R-K/R-L-L-E-G-E-E/D-X-R-L/N/I [4]. Multiple alignments using type VI IF proteins as well as desmin and NF-M sequences show that the helix termination motifs of type VI IF chains diverge from the consensus sequence at specific sites: **D/G**/E-**R/D/G/**Y-Q-X-L-**A/M/**L-**H/Q**-**L/**V-K-X-**S/G**-L-**S-**X-E-**V**-A-T-Y-R-**T/S/A**-L-L-E-**A/**G-E-X-R-L/I/**Q/E **(Fig. 2A). The helix termination motif contains key residues that are important for structure and assembly of IFs and this region represents one of the two mutation hotspots in IF proteins [40]. The observed divergences suggest that type VI IF proteins evolved in a branch distinct from NF proteins and that they gained new residues at the same sites of segment 2B: Q95, H98, S102, S104 and A116 (asterisks in Fig. 2A). Those residues may be implicated in specific functions of type VI IF proteins.

To investigate potential relationships among type VI IF proteins, a phylogenetic tree was constructed using a multiple alignment of the entire sequence of known type VI proteins. The sequence of NF-M proteins was also used to examine how closely related type VI IF proteins are to NF proteins. As seen in Fig. 2B, such phylogenetic analysis shows that synemin, transitin, tanabin and nestin are all part of a group that is distinct from the NF-M protein. This correlates with the idea that type VI IF genes are the evolutionary result of a duplication event of an ancestral NF gene and that type VI IF genes then evolved independently from NFs. Transitin and tanabin are probably more closely related to each other than to other type VI IF proteins as suggested by their close phylogenetic proximity. In summary, our analysis indicates that tanabin, transitin and nestin form a branch distinct from NF proteins and they are more closely related to each other than to synemin.

### 2-Tanabin, transitin and nestin are orthologous proteins

**Figure 3:**
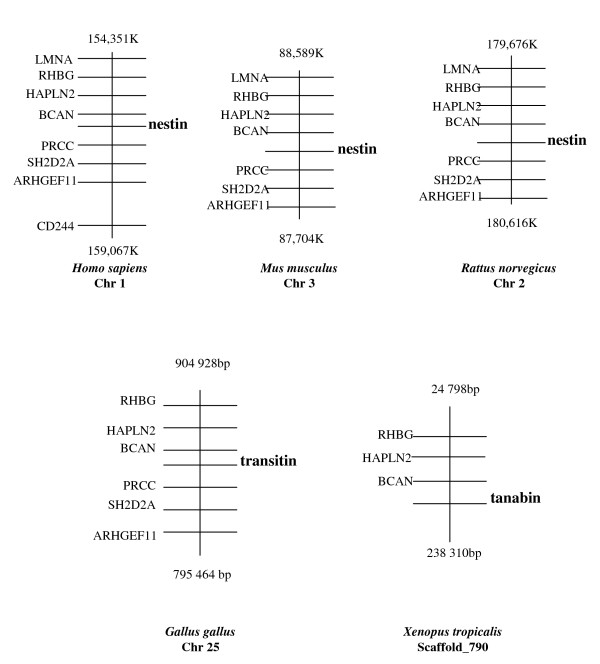
**Syntenic relationship among frog tanabin, avian transitin and nestin from human, mouse and rat**. Physical maps of human, mouse, rat and chicken genomic sequences along with an anuran scaffold (*X. tropicalis *scaffold_790) were used to identify genes neighboring nestin, transtin and tanabin. Each diagram represents a chromosomal region whose position is defined by the number of base pairs from the telomere of the short arm of the chromosome (top and bottom of each diagram). Each horizontal line represents a gene position on the chromosome. Nestin, transitin and tanabin genes are shown in bold for clarity. Diagrams are arranged in such a way that the gene positions are visualized in the same orientation. LMNA, lamin A/C; RHGB, Rhesus blood group, B glycoprotein; HAPLN2, hyaluronan and proteoglycan linked protein 2; BCAN, brevican; PRCC, papillary renal cell carcinoma; SH2D2A, SH2 domain protein 2A; ARHGEF11, Rho guanine nucleotide exchange factor (GEF) 11 and CD244, CD244 natural killer cell receptor 2B4.

To investigate whether tanabin, transitin and nestin are orthologous proteins, a synteny analysis was performed using the NCBI mapped genomic scaffolds of human, mouse, rat and chicken along with the JGI genomic scaffolds of *X. tropicalis*. As seen in figure 3, regions of strong synteny conservation exist around both the nestin gene in all three mammalian species and the transitin gene in chicken. Nestin and transitin genes both locate between BCAN and PRCC genes and the order of the neighboring genes is conserved. Tanabin was located close to the BCAN gene on *X. tropicalis *Scaffold_790. The PRCC, SH2D2A and ARHGEF11 genes were grouped on a different scaffold (not shown). This indicates that the genomic location of amphibian tanabin, avian transitin and mammalian nestin has been conserved. These observations, combined with the fact that these genes have the same intron distribution strongly suggest that tanabin, transitin and nestin originate from the same ancestral gene.

### 3-ATPase and GTPase activity of the transitin HR domain

We examined the domain architecture of the type VI IF proteins through the CDD database (Table 2). As expected, this analysis confirmed the presence of a canonical IF domain in the N-terminal moiety of tanabin, transitin and nestin but a new domain was revealed in their C-terminal extremity, corresponding to an ATPase motif of either the SMC type (Structural Maintenance of Chromosomes superfamily of proteins) or the AAA+ family (ATPases Associated with various cellular Activities). The highest score was obtained for the transitin tail and corresponded to its HR domain. Lower scores were obtained for tanabin and nestin C-terminal extremities from different species (Table 2). The exact function of the transitin HR domain is unknown. Paranemin, a splice variant of transitin, has been shown to be involved in the formation of an extended IF network when co-transfected with desmin in IF-free cells [23]. The HR domain may be implicated directly in this mechanism since this protein motif has been shown to interact with type III IF proteins vimentin and desmin during myogenesis (Guérette *et al*., submitted). The low sequence identity between the repeated motifs of transitin and nestin carboxy-terminal extremities and the lowest score for an ATPase motif in the nestin tail may be the consequence of the rapid evolution of this part of the gene. As previously shown, the carboxy-terminal region of nestin contains a repeated domain subjected to size fluctuations among rodents and human that could be linked to its higher evolutionary rates, compared to the IF rod domain [8]. Incidentally, better E-values were obtained for an ATPase motif using sequences from dog, dolphin and human rather than from rodents (Table 2).

**Table 2:** ATPase score motif associated with the C-terminal tail of tanabin, transitin and nestin of different species by Conserved domain database analysis on the NCBI server. An ATPase motif was detected in tanabin, transitin and nestin in comparison with the domain architectures available in the Conserved domain database on the NCBI server. The score shown in the table is calculated using PSSM scoring matrix based on protein alignment. The expected value is also given for each alignment and significant E-values for this database searching are considered to be > 10-5. Protein sequences for this analysis were obtained from sequenced genomes available on NCBI and JGI genome assembly v4.1. Tanabin-xt: 186291, chicken transitin: X80877, dog predicted-nestin: XP_547531.2, mouse nestin: NP_057910.3, rat nestin: NP_037119.1 and human nestin: NM_006617

**Query sequence**	**E-value (IF domain)**	**E-value (ATPase domain)**
Tanabin (*Xenopus laevis*) gi|549051	2e-15	0.0003^1^
Transitin (*Gallus gallus*) gi|45384298	5e-14	5e-15^2^
Nestin^3 ^(*Canis familiaris*) gi|73961547	6e-8	2e-8^1^
Nestin^3 ^(*Monodelphis domestica*) gi|126307847	3e-19	0.000009^1^
Nestin^3 ^(*Bos taurus*) gi|76612380	9e-13	No hits
Nestin (*Rattus norvegicus*) gi|6981262	7e-11	0.008^1^
Nestin (*Mus musculus*) gi|50363232	0.000002	0.003^2^
Nestin^3 ^(*Macaca mulatta*) gi|109017378	9e-13	No hits
Nestin (*Homo sapiens*) gi|1346682	9e-7	0.00004^2^

^1 ^NCBI imported model COG5271, MDN1, AAA ATPase containing von Willebrand factor type A domain

^2 ^NCBI imported model COG1196, Smc, Chromosome segregation ATPases

^3 ^Predicted sequence

**Figure 4:**
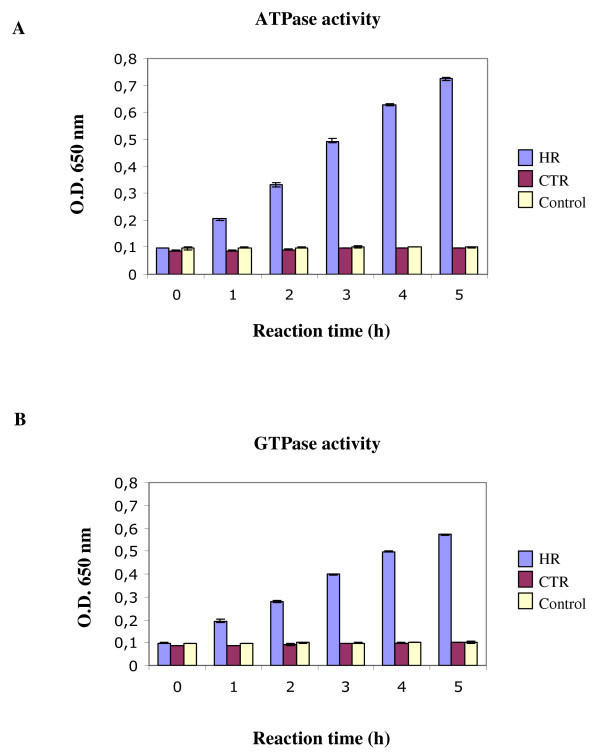
***In vitro *ATPase and GTPase activity of the HR domain**. The HR domain of chicken transitin and the CTR domain of mouse nestin were expressed in bacteria and purified. Their ATPase/GTPase activities were tested individually using the malachite green colorimetric method for phosphate release determination. Phosphate release was determined at 25°C for each recombinant protein incubated with either **A) **ATP or **B) **GTP. The optical density at 650 nm was determined in triplicate at regularly timed intervals during the course of the enzymatic reaction and the mean values are presented with standard deviation error bars. Control: Buffer with the HR recombinant protein without NTP.

We verified by *in vitro *means whether the C-terminal repeated domains of both chicken transitin and mouse nestin could have either ATPase or GTPase activity. To do so, fusion proteins representing either the entire HR domain of transitin (HR) or the entire C-terminal repeated domain of nestin (CTR) were expressed in the same bacterial strain. These fusion proteins were purified and ATP and GTP hydrolysis activities were assayed for phosphate release using the malachite green colorimetric method for phosphate determination (Fig. 4) [41]. The HR domain of transitin has ATPase (Fig. 4a) as well as GTPase (Fig. 4b) activity as shown by an OD augmentation in our assay. Using such an assay, it has not been possible to detect any ATPase or GTPase activity in the mouse nestin CTR. These experimental results are concordant with our *in silico *analysis suggesting that transitin ATPase activity could have been lost during the rapid evolution of nestin C-terminal repeat domain in mammals.

The SMC and AAA+ proteins contain two nucleotide-binding modules, the Walker A and Walker B motifs [42], defining a broad superfamily of nucleotide-binding proteins including many ATPases, myosin and numerous kinases [43]. In these proteins, the ATP-binding module is activated by the formation of an oligomeric assembly and drives conformational changes affecting target substrates. The Walker A and B motifs of SMC proteins, which show the highest score with transitin HR domain, are located in the N- and C-terminal extremities of these proteins and are separated by a central domain composed of a hinge sequence flanked by two long coiled-coil motifs [44]. As the bulk of transitin HR domain is predicted to have a coiled-coil structure [45], it may be anticipated that the HR domain presents ATPase activity at one or both ends. The Walker B signature motif, as decribed by Walker [42], corresponds to R/KX_3_GX_3_Lh_4_D (h = hybdrophobic and X = any residue) and could loosely match with the sequence **R**DLQE**G**HGD**L**QVEHE**D **located at the N-terminal extremity of the HR domain. In fact, hydrolytic activity has been detected in our ATPase assay using fragment HR_1–4_, which contains the first 4 repeats of the HR domain. In addition, this domain has been shown to completely disassemble the IF network when overexpressed in avian myoblasts (Guérette *et al*., submitted). Site-directed mutagenesis is now under way to identify the most important residues for ATP binding and hydrolysis.

### 4-Conservation of the HR domain in some species

**Figure 5:**
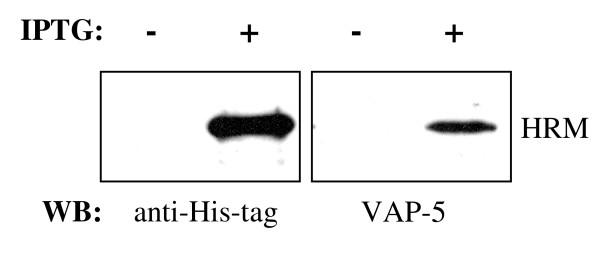
**Reactivity of the HR-specific monoclonal antibody VAP-5 with the "hypothetical" mouse protein HRM**. A fragment of mouse genomic clone RP2389A3 (gi: 106520665) showing the highest sequence identity to chick transitin HR domain has been subcloned in a pET-based expression vector and a His-tag-HRM fusion protein bacterially produced under IPTG induction. A co-migrating immunoreactive band was observed in Western blots using the anti-His-tag antibody and VAP-5 in extracts of bacteria submitted to IPTG induction but was not detected in the uninduced culture.

Since the HR domain is a feature unique to transitin in that it possesses both ATPase and GTPase activities *in vitro*, we focused on the evolution of this particular domain. Assuming that transitin and nestin are orthologous proteins and that nestin CTR does not show significant sequence identity with transitin HR domain, we looked for the presence of HR domain-like sequences in other genomic regions. BLASTn searches demonstrated that the transitin HR domain has sequence similarities to two genomic clones from human and mouse origins. The human clone RP1 155d22 (gi: 2827470) is located on chromosome 6q27 and is 82% identical over 118 nt to the HR domain of chicken transitin (E-value = 4e-7). This clone does not correspond to the human nestin gene, located on chromosome 1. The mouse genomic clone RP2389A3 (gi: 106520665) is located on chromosome 17 and is 79% identical over 353 nt to the HR domain of chicken transitin (E-value of 5e-16). An hypothetical protein is also predicted (gi: 94403328) with an E-value of 4e-13. This protein is encoded in part by an RP2389A3 clone that likely corresponds to the mouse version of the HR domain. The human and mouse versions of the HR domain are not part of any currently known gene. Moreover, these regions are devoid of any IF core feature in the 5' regions suggesting that the putative HR domain found in human (HRH) and mouse (HRM) may be part of proteins that are not members of the IF family. The HR domain of chicken transitin was used as a query for a BL2SEQ alignment with the HRH and HRM sequences. Both have 50% protein sequence identity with the chicken transitin HR domain. Consensus repeated sequences found in HRH and HRM are LQVEEGS and MQVEHDG respectively, compared with the consensus sequence of LQVEHGD in the chicken transitin HR domain. Furthermore, monoclonal antibody VAP-5 directed against a repeated epitope of the HR domain of chick transitin [45] targets a similar epitope on a synthetic HRM protein (Fig. 5). The HRM sequence has been subcloned in a pET-based expression vector and a His-tag-HRM fusion protein bacterially produced under IPTG induction. In induced bacterial cultures, an immunoreactive band of the expected Mr value was observed in Western blots using the anti-His-tag antibody and VAP-5. This protein was not detected in uninduced cultures (Fig. 5: without IPTG).

**Figure 6:**
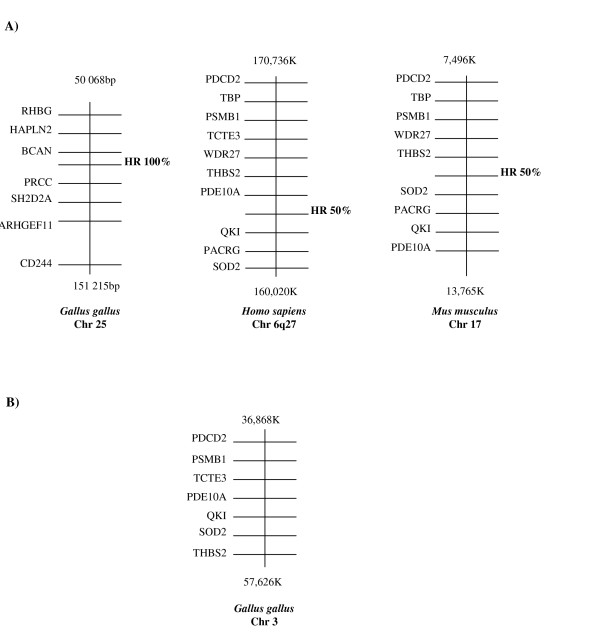
**Syntenic relationships of the conserved HR domain in birds, humans and mice**. Physical maps of human, mouse, rat and chicken genomic sequences were used to identify genes neighboring the conserved HR domains. Each diagram represents a chromosomal region whose position is defined by the number of base pairs from the telomere of the short arm of the chromosome (top and bottom of each diagram). Each horizontal line represents the position of a gene and is placed in physical order on the chromosome. Diagrams are depicted in such a way that genes are oriented in the same way. **A) **The HR domains were syntenic in human (chromosome 6q27) and mouse (chromosome 17). They share 50% identity with the chicken HR domain of transitin located on chromosome 25 (see Fig. 3). **B) **A corresponding gene cluster was found on chicken chromosome 3 from which an "avian" HR domain genomic segment was absent. RHGB, Rhesus blood group, B glycoprotein; HAPLN2, hyaluronan and proteoglycan link protein 2; BCAN, brevican; PRCC, papillary renal cell carcinoma; SH2D2A, SH2 domain protein 2A; ARHGEF11, Rho guanine nucleotide exchange factor (GEF) 11 and CD244, CD244 natural killer cell receptor 2B4; PDCD2, programmed cell death 2; PSMB1, proteasome (prosome, macropain) subunit, beta type, 1; TCTE3, t-complex-associated-testis-expressed 3; PDE10A, phosphodiesterase 10A; QKI, quaking homolog; SOD2, superoxide dismutase 2; THBS2, thrombospondin 2, PACRG, PARK2 co-regulated and WDR27, WD repeat domain 27.

A synteny search was conducted using the physical maps of human, mouse and chicken genomes to determine whether the genes encoding the HR domain in human and mouse were conserved among the same set of neighboring genes. The "mammalian" HR domain genomic segments were syntenic in human (chromosome 6q27) and mouse (chromosome 17) (Fig. 6a) and correspond to a well-conserved gene cluster on chicken chromosome 3 from which the "avian" HR domain genomic segment was absent (Fig. 6b) as it is located on chromosome 25 as part of the transitin gene (Fig. 6a). These observations suggest that the nucleotide sequence corresponding to the conserved HR domain in human and mouse likely originated from the exon coding for the HR domain in a chicken transitin ancestral gene.

To establish whether sequences similar to the HR domain of chicken transitin could be found in species located in upstream branches of vertebrate evolution, a tBLASTn search of *Takifugu rubripes *(pufferfish) was made using the entire transitin protein sequence. Genome sequence analysis of *T. rubripes *did not reveal the presence of a nestin homolog [38]. This may explain why the rod domain of desmin was found by tBLASTn analysis to be the strongest hit to the rod domain of transitin (27% identity). On the other hand, the HR domain of transitin is 22% identical to a retinitis pigmentosa GTPase regulator-like (RPGR-like) protein spanning 555 nt with an E-value of 2e-28. As the protein sequence of tanabin is closely related to transitin, tanabin was used to conduct a second tBLASTn search. Once again, the RGPR-like protein emerged (22% identical) spanning 580 nt with an-E value of 2e-21 to the C-terminal of tanabin. The observations suggest that tanabin and transitin tail domains have evolved from an RPGR-like protein.

### 5-Model for the evolution of type VI IF proteins

**Figure 7:**
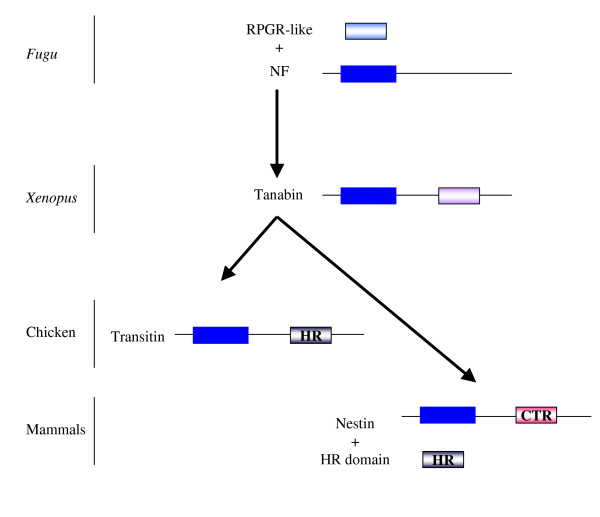
**Evolutionary model for type VI IF proteins**. *Fugu *lacks obvious nestin homologs [38] and NF proteins may well represent ancestral type VI IF proteins [8]. Our model proposes that the first type VI IF protein resulted from the fusion of a RPGR-like cassette C-terminal to a NF gene. The C-terminal domain of a tanabin-like protein may have evolved giving rise to the HR domain displaying nucleotide hydrolysis activity in birds whereas this activity was progressively lost in mammalian nestins. In addition, the HR domain may have been duplicated and conserved in a mammalian gene that is not part of the IF family.

Type VI IF proteins have two of the three intron positions in common with type IV NF genes but the level of similarity in the α-helical regions is only 20% compared with 50% observed among the NF genes. It has been postulated that a type VI IF gene ancestor branched off before the split into the three NF genes where the ancestor later gained a third intron [8]. We propose a model to explain the evolution of type VI IF proteins (Fig. 7). This model suggests that the first type VI IF gene arose evolutionarily as the result of incorporation of an RPGR-like cassette into the 3' extremity of an ancestral NF gene. From tanabin in amphibians, the history of type VI IF genes may have included loss of the supplementary intron in the 3' part of the gene followed by fast evolution of the C-terminal RPGR-like cassette giving rise to the HR domain as it is found in avian transitin. In mammals, this domain must have evolved quickly as some similarity is found between the chicken HR domain and the dog CTR domain but none exists between the chicken HR domain and the CTR domain of mouse and human nestin. According to this model, the tail domain of human nestin would not have resulted from the loss of the RPGR-like cassette and its substitution by the CTR domain but rather from a fast evolution rate leading to loss of its nucleotidase activity. In addition, the HR domain could have been duplicated since it is found in a non-IF hypothetical gene which has been conserved in a syntenic way among mice and humans.

## Conclusion

Many lines of evidence based on sequence identity, gene structure, synteny comparison and phylogeny searching point to the conclusion that frog tanabin, chicken transitin and mammalian nestin are orthologous members of the type VI IF proteins. The C-terminal domains of both tanabin and transitin were predicted to have nucleotide hydrolysis activity *in silico*, and indeed, ATPase activity was measured *in vitro *within the HR domain of transitin. This domain apparently experienced a fast evolution rate that could have resulted in loss of ATPase activity in mammals.

## Methods

### Known type VI protein sequences

Type VI IF protein sequences used in this study were: chicken transitin [GenBank: X80877], human nestin [GenBank: NM_006617], tanabin-xl [GenBank: M99387], tanabin-xt [JGI: 186291], human synemin [GenBank: CAC83859]. Other protein sequences used in this study were: human NF-M [GenBank: CAA68276], mouse NF-M [GenBank: CAA29127], human desmin [GenBank: NP_001918], mouse desmin [GenBank: NP_034173], and RPGR-like [GenBank: AAG00554].

### BLAST, multiple sequence alignment and phylogenic tree analysis

BLASTn, BLASTp, tBLASTn and BL2SEQ searches were performed in NCBI or JGI (in the case of *X. tropicalis*) databases. Default parameters were used to conduct the searches. Multiple sequence alignments were prepared using CLC workbench version 3.0.1. These multiple sequence alignments were used to create a phylogenic tree with neighbor-joining methods with 100 bootstrap analysis using CLC workbench version 3.0.1 software.

### Syntenic relationship identification

Chromosomal locations of nestin and transitin genes were identified using physically-mapped human (build 36.1), mouse (build 35.1), rat (build v3.4) and chicken (build 1.1) genomes. For each species, syntenic genes were located using mapviewer (NCBI). In the case of transitin, as this gene was not located on any chicken chromosome (build 1.1) at the onset of our study, the contig NW_094723.1 containing the transitin gene was used to identify neighboring genes. These genes were later located to chicken chromosome 25 (build 2.1) and then compared with physically mapped human, mouse and rat genomes. To analyze synteny relationships of the transitin HR domain, the contigs containing the human and mouse HR sequences were located on human and mouse chromosomes and the genes surrounding these sequences were positioned and compared with the chicken genome.

### Assay of ATPase and GTPase activity

Different fusion proteins were cloned as described previously [45]. Fusion proteins were purified by T7-tag affinity purification (Novagen) or by His-tag Sepharose (Amersham) according to the instructions of the manufacturers. ATPase and GTPase activities were determined using "malachite green phosphate assay kits" from BioAssay Systems. After purification, fusion proteins were dialyzed against Buffer A (50 mM Tris-HCL pH 7.5, 10 mM MgCl_2_, 100 mM NaCl, 20 mM KCl and 1 mM β-mercaptoethanol) overnight at 4°C and concentrated using Centricon YM-10 (Millipore). Protein concentrations were determined with a Micro BCA Protein Assay kit (Pierce). Fusion proteins (0.04 μg/μl) were incubated in Buffer A with 1 mM ATP or 1 mM GTP at 25°C. Aliquots were taken at different times and mixed with malachite green buffers as described by the manufacturer (BioAssay Systems). After 15 min of incubation, the O.D. at 650 nm was determined using a Multiskan Spectrum spectophotometer (ThermoLabSystems). A standard curve with free phosphate was produced according to the instructions of the manufacturer.

### SDS-PAGE and Western blots

A fragment of mouse genomic clone RP2389A3 (gi: 106520665) showing the highest sequence identity to chick transitin HR domain has been amplified by PCR and subcloned in a His-tag containing pET30 expression vector to transform BL21(DE3)pLysS bacteria. The expression of the His-tag-HRM fusion protein was induced by 0.1 mM IPTG and bacterial pellets directly solubilized in electrophoresis sample buffer. The protein samples were resolved by SDS-PAGE and transferred to nitrocellulose membranes as described [46]. For Western blots, the membranes were saturated for 1 hour at room temperature using 1% blocking reagent (Roche Diagnostics). The primary antibody (anti His-tag or mAb VAP-5) was incubated for 1 hour and the secondary antibody for 45 minutes, both at room temperature. The proteins were detected using the BM chemiluminescence kit (Roche Diagnostics).

## Authors' contributions

Bioinformatic and experimental analysis were performed by DG. All authors contributed to designing experiments, analyzing data and writing the manuscript. They all accepted the final version.
